# Asymptomatic Carriage of *C. botulinum* Type D/C in Broiler Flocks as the Source of Contamination of a Massive Botulism Outbreak on a Dairy Cattle Farm

**DOI:** 10.3389/fmicb.2021.679377

**Published:** 2021-06-29

**Authors:** Rozenn Souillard, Daniel Grosjean, Thibault Le Gratiet, Typhaine Poezevara, Sandra Rouxel, Loïc Balaine, Sabrina Macé, Laure Martin, Fabrizio Anniballi, Marianne Chemaly, Sophie Le Bouquin, Caroline Le Maréchal

**Affiliations:** ^1^ANSES, French Agency for Food, Environmental and Occupational Health Safety, Epidemiology, Health and Welfare Unit, Ploufragan, France; ^2^DDCSPP de la Meuse, Departmental Authority in Charge of Veterinary Services for Meuse Department, Bar-le-Duc, France; ^3^ANSES, French Agency for Food, Environmental and Occupational Health Safety, Hygiene and Quality of Poultry and Pig Products Unit, Ploufragan, France; ^4^Department of Veterinary Public Health and Food Safety, National Reference Centre for Botulism, Istituto Superiore di Sanità, Rome, Italy

**Keywords:** botulism, cattle, poultry, epidemiology, hatchery, investigation, MLVA, PCR

## Abstract

In winter 2018, a massive type D/C cattle botulism outbreak occurred on a mixed dairy and broiler farm in France. An investigation was conducted based on the hypothesis of asymptomatic carriage in poultry. We set out to identify the source of contamination of the dairy cattle and to monitor the contamination of broilers over time, including the hatchery delivering chicks to the farm. Environmental samples were collected on the farm during the cattle outbreak (*n* = 40), after the outbreak for three successive broiler flocks (*n* = 128), and once in the hatchery delivering the chicks (*n* = 58). These samples were analyzed using real-time PCR after an enrichment step to detect *Clostridium botulinum* type D/C. The results showed contamination in the manure from the broilers raised just before the onset of the cattle outbreak (5 + /5), as well as in some of the components of the cattle ration (3 + /17). This latter contamination is likely due to the use of the same tractor bucket to remove litter from the poultry house and to prepare the cattle ration on the same day. Contamination monitoring over several months revealed continuous asymptomatic carriage in the broilers (4 + /20 and 17 + /20 cloacal swabs in 2 successive flocks), a persistence of *C. botulinum* type D/C in the ventilation system of the poultry house (8 + /14), and contamination of the equipment coming from the hatchery used for delivering the chicks (3 + /18). Further investigations conducted in the hatchery demonstrated contamination in the hatchery by *C. botulinum* type D/C (6 + /58). Comparison of samples using a multilocus variable number tandem repeat analysis showed the same profile for samples collected on broilers, cattle and in the hatchery. This study highlighted the crucial role of the implementation of biosecurity measures in mixed farms to avoid cross-contamination between production units given the potential asymptomatic carriage of poultry. This study also revealed the contamination of the poultry hatchery. Further investigations are required to better understand the role of hatcheries in the epidemiology of animal botulism.

## Introduction

Botulism is a severe neurological disease caused by botulinum neurotoxins (BoNT) that prevent the release of acetylcholine at synaptic junctions and result in progressive symmetrical flaccid paralysis of muscles. There are nine different BoNTs (A, B, C, D, E, F, G, H, or H/A, or F/A, X) (Rasetti-Escargueil et al., 2020) and more than 40 subtypes have been described (Peck et al., 2017). Human botulism is a rare disease is mainly caused by BoNTs A, B, E, and to a lesser extent, F (Rasetti-Escargueil et al., 2019). Botulism is more common in animals than in humans and results in high mortality rate, raising significant animal welfare and economic concerns (Anniballi et al., 2013; Relun et al., 2017; Rasetti-Escargueil et al., 2019). Avian botulism is generally associated with BoNT C/D, whereas bovine botulism is more frequently associated with BoNT D/C, and to a lesser extent, BoNT C (Woudstra et al., 2012; Bano et al., 2017; Le Gratiet et al., 2020). Considering the serious consequences of botulism on bovine and avian species, a better understanding of this disease — particularly in terms of potential mechanisms of transmission — is crucial to improve prevention and management of animal botulism outbreaks in an efficient manner.

Poultry litter has been considered as a major source of contamination for cattle botulism outbreaks via contact or close proximity (Popoff, 1989; Payne et al., 2011; Relun et al., 2017). Cross-contamination from poultry to cattle has been widely reported in the literature (Senturk and Cihan, 2007; Payne et al., 2011; Ramírez-Romero et al., 2014; Relun et al., 2017; Souillard et al., 2017). Poultry is indeed considered as a reservoir and source of amplification of type D *Clostridium botulinum* and its toxin (Popoff and Argente, 1996). Poultry litter can be used as fertilizer, animal bedding or even as feed supplements (Payne et al., 2011). Surprisingly, no data is available on the prevalence of *C. botulinum* or BoNTs in poultry litter or more generally in healthy poultry. Recently, healthy carriage on a poultry farm was suspected as a source of two cattle botulism outbreaks due to a transfer of poultry manure to the cattle farms (Souillard et al., 2017). Consequently, asymptomatic carriage of *C. botulinum* in poultry can occur, which may represent a reservoir of *C. botulinum* (Rasetti-Escargueil et al., 2019). Animals are either resistant to some BoNT types or *C. botulinum* carriage occurs at low bacterial loads in the digestive tract (Rasetti-Escargueil et al., 2019). These low levels may explain the failure to detect *C. botulinum* carriage in previous studies, as they may be below the limit of detection of available methods (Popoff, 1989).

In 2018, a large BoNT type D/C cattle botulism outbreak occurred on a farm with both dairy and broiler production units in eastern France. Based on the hypothesis of asymptomatic *C. botulinum* carriage in poultry, the objectives of this study were (i) to identify the source of cattle botulism contamination using epidemiological investigations and strain tracking and (ii) to monitor the contamination of broilers over time including at the hatchery delivering chicks to the farm.

## Materials and Methods

### Case History and Diagnosis of Botulism on the Farm

**FIGURE 1:**
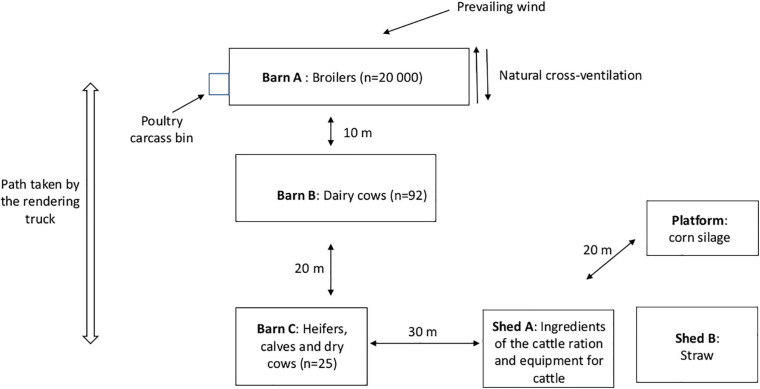
Layout of the mixed farm site that experienced a type D/C botulism outbreak in Eastern France in winter 2018.

A botulism outbreak occurred in winter 2018 on a farm with dairy and broiler production in Meuse department, located in eastern France. Figure 1 shows the different barns of the farm. Ninety-two dairy cows were being housed in Barn B and 25 heifers, calves and dry cows in Barn C. These two barns are separated by about 20 m. During winter, cattle are kept indoors and, beginning early spring, grazed on pasture. The Barn A poultry house is separated by about 10 m from Barn B. It is a “Louisiane”-style barn of 1000 m^2^ with windows, natural and transversal ventilation, and a dirt floor. In this house, a flock of 20 000 broilers (Flock No. 1) was reared in January 2018. Moreover, the farm has two sheds: Shed A for the storage of the cattle ration ingredients and Shed B for straw storage. In addition, corn silage is also stored on a platform near Shed B. A second site for cattle production is located about 10 km away from this area.

On January 29, the first clinical signs of paralysis were observed in dairy cows in Barn B and mortality started (Table 1). On 30 January, samples were collected on two cows that died after showing clinical signs suggestive of botulism. The liver, gall bladder, rectal contents, ruminal contents, feces, and intestines were frozen before shipment to the laboratory. *C. botulinum* types C, D, C/D, and D/C in these samples were screened for as previously described (Le Maréchal et al., 2019). Ruminal contents from the two investigated cows as well as rectal contents from one cow were positive for *C. botulinum* type D/C using real-time PCR after enrichment in trypticase peptone-glucose-yeast extract (TPGY) broth. None of the samples were positive when using fortified-cooked meat medium (F-CMM) with a thermal treatment (70°C, 10 min). On 31 January, signs of paralysis and mortality also occurred in the heifers in Barn C. No clinical signs and no mortality were observed on the second site 10 km away. Vaccination using Ultravac^®^ Botulinum (Zoetis, France) was performed on February 2 with a second injection on March 5 in both cattle barns.

**TABLE 1:** Chronology of the events on the farm affected by the cattle botulism outbreak type D/C and detection of *C. botulinum* type D/C during the outbreak.

	22/01/18	26/01/18	27-28/01/18	29/01/18	30/01/18	31/01/18	02/02/18	05/02/18	23/02/18	05/03/18
Chronology of the outbreak events	Departure of broilers (Flock No 1 Barn A) for slaughter	Removal of broiler litter using a tractor bucket and storage of manure in a field Transfer of the ingredients of the cattle ration (Shed A) with the same bucket in a mixing wagon to feed cows.	Heifers fed with leftovers from the cattle ration Cleaning and disinfection of the broiler house (Barn A)	Detection of paralysis and first cow mortalities (Barn B)	Diagnosis of botulism: *C. botulinum* type D/C in ruminal contents **(S3)** (2 + /2)* and rectal content (1 + /2)*	Paralysis and heifer mortalities (Barn C)	Vaccination of all animals (including calves, dry cows) with Utravac Botulinum (first injection)			Vaccination with Ultravac Botulinum (second injection)

Detection of *C. botulinum* type D/Con the farm* (n = 40)					Cattle ration Shed A (*n* = 3) Wrapped grass 1 + /3			**Cattle ration** **Shed A (*n* = 11)** Meslin (S1) **1 + /3** Rape **1 + /3** Brewery grains 0 + /3 Corn 0 + /2 **Cattle ration** **Platform (*n* = 3)** Corn silage 0 + /3 **Broiler manure** **stored since 26/01 (*n* = 5)** Manure (S2) in a field **5 + /5**	**Barn B-(*n* = 3)** Liquid manure **1 + /1** Swab of the floor of the stall **1 + /1** Swab of cattle feed table 0 + /1 **Shed A (*n* = 5)** Swab of the floor of the shed 0 + /1 Swab of the bucket **1 + /1** Swab of the tarpaulin covering rape **1 + /1** Swab of the rape storage area 0 + /1 Swab in the mixing wagon **1 + /1** **Barn A (n = 10)** *Inside the house* Swab of the ventilation system **2 + /4** Swab of the floor of the house 0 + /1 Swab of the shower? room 0 + /1 Feed from silo 0 + /1 *Outside the house* Swab of the surroundings 0 + /1 Swab of the carcass bin **1 + /1** Swab of the path 0 + /1	

*Number of positive samples/number of samples collected.

**FIGURE 2:**
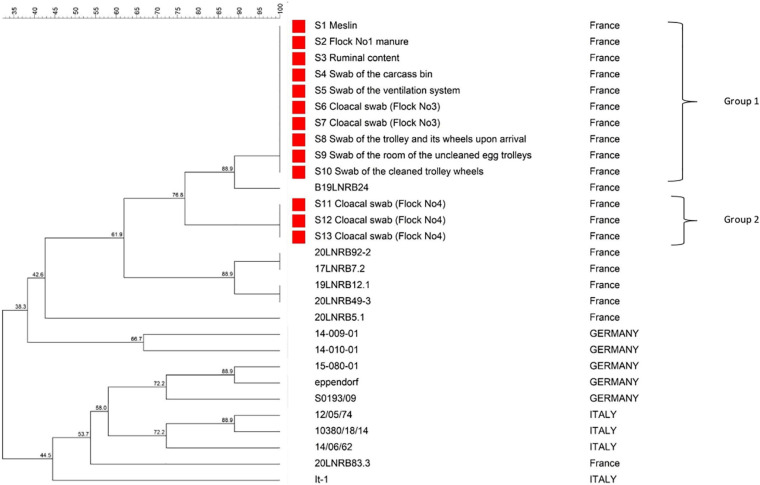
Multilocus variable-number tandem repeat analysis (MLVA) dendrogram of 13 samples collected in this study and positive for *C. botulinum* type D/C (in red) and 16 other strains of *C. botulinum* type D/C (typed in other studies) constructed using the UPGMA method, implemented in BioNumerics. The name and code of samples from our study and names of strains from previous studies and their respective country are shown on the right. Samples S1–S13 were separated in 2 groups based on MLVA similarities.

*Samples tested by MLVA are underlined **and the sample code in**Figure 2 is indicated in **parentheses**. Samples detected positive are in bold.*

Two phases of death were observed on the farm. Mortality was high within the first week (January 29 to February 5) with 68 dead cows in Barn B and 8 heifers in Barn C. A second mortality phase was observed from February 26 to March 5 with 15 cows and 1 heifer. Out of the 117 cows present on the farm at the beginning of the outbreak, 92 cows died, indicating a mortality rate of 79%. All animals with clinical signs eventually died (naturally or humanely euthanized), no recovery was reported.

### Epidemiological Investigation and Collection of Environmental Samples on the Farm

An epidemiological investigation was conducted on the farm. A questionnaire was filled out with the farmer and the veterinarian to describe the history and chronology of the cases. It included questions on the overall management of cattle and poultry (farming practices and manure management, etc.) and the movements of material, personnel, and vehicles between the poultry and dairy production areas.

During the outbreak, environmental samples (*n* = 40) were collected on the farm on three different days (January 30, February 5, and February 23) to investigate the source of contamination of the cattle (Table 1): 17 samples of the cattle ration stored in Shed A (*n* = 14) and on the platform (*n* = 3), 5 samples of the broiler manure from Flock No. 1 stored in a field since January 26, 3 samples in Barn B, 5 samples in Shed A and 10 samples in Barn A and its surroundings.

After the end of the outbreak, samples (*n* = 128) were also collected for several months in the poultry house to monitor the contamination of broilers over time (Table 2): 9 swabs after cleaning and disinfecting the house with 7 swabs inside the house and 2 swabs outside the house; 55 swabs on the day of chick delivery to the farm, including 11 swabs inside the house; 2 swabs outside the house, 20 cloacal swabs on chicks, 18 swabs on hatchery-origin material, and 4 swabs on the material returned to the hatchery; and finally 64 swabs at the end of the rearing period, with 4 swabs inside the poultry house and 60 cloacal swabs of broilers on the three following broiler flocks (Flocks No. 3, 4, and 5). It was not possible to sample the second flock of broilers reared just after Flock No. 1. Swabs used for sample collection in the farm were obtained from Sodibox (Nevez, France). Cloacal swabs were collected by veterinarians in accordance with the European and French regulation on farmed animal protection.

**TABLE 2:** Detection of *C. botulinum* type D/C in the poultry house after the cattle outbreak (*n* = 128) using swab samples.

		After extensive disinfection of the poultry house *n* = 9		Upon chick delivery *n* = 55			Upon broiler departure *n* = 64		
Flock No 1*	21/03/18	Inside the house *n* = 7	Ventilation system (S5) **2 + /4** Floor of the house 0 + /1 Changing room 0 + /1 Feed silo 0 + /1						
		Outside the house *n* = 2	Carcasses bin (S4) **1 + /1** Surroundings 0 + /1						

Flock No 2*						ND			ND

Flock No 3*				22/05/18	Inside the house *n* = 7	Ventilation system **2 + /4** Floor of the house 0 + /1 Changing room 0 + /1 Feed silo 0 + /1	13/06/18	Inside the house *n* = 1	Floor 0 + /1
					Outside the house *n* = 2	Surroundings 0 + /1 Carcasses bin 0 + /1		Broilers *n* = 20	Cloacal swabs **4 + /20** (S6 and S7)

Flock No 4*				9/07/18	Inside the house *n* = 2	Ventilation system 0 + /2	6/08/18	Inside the house *n* = 3	Ventilation system **2 + /2** Floor **1 + /1**
					Material from the hatchery *n* = 2	Chick box bottom **2 + /2**		Broilers *n* = 20	Cloacal swabs **17 + /20** (S11 to S13)

Flock No 5*				27/08/18	Inside the house *n* = 2	Ventilation system **2 + /2**	24/09/18	Broilers *n* = 20	Cloacal swabs 0 + /20
					Material from the hatchery *n* = 16	Chick box bottom 0 + /2 Article of the box bottom 0 + /10 10 chick boxes upon arrival 0 + /2 Trolley and its wheels upon arrival **1 + /2** (S8)			
					Chicks from the hatchery *n* = 20	Cloacal swab on chicks 0 + /20			
					Material returned to the hatchery *n* = 4	10 chick boxes upon departure 0 + /2 Trolleys and their wheels upon departure 0 + /2			

ND, no data.

*Number of positive samples/number of samples collected (positive samples are in bold).

*Samples tested by MLVA are underlined **and the sample code in**Figure 2 is indicated in **parentheses.***

### Epidemiological Investigation and Collection of Environmental Samples in the Hatchery

An epidemiological investigation was also conducted at the hatchery providing the chicks. A questionnaire was filled out with the hatchery owner to collect information on the hatchery operations (from egg hatching to the departure of chicks, cleaning and disinfection, waste management, and vehicle movements on the site, biosecurity measures, etc.). A breeder house was also located on a site near the hatchery. At the hatchery site, samples (*n* = 58) were collected (swabs taken on the walls, floor and equipment): 41 inside the hatchery (egg receiving room, incubation and hatcher rooms, chick sorting, and departure rooms), 9 outside the hatchery (truck platform, waste containers, central area and breeder house surroundings) and 8 in annex rooms (trolley storage rooms and refrigerated egg holding rooms).

### Culture Conditions, DNA Extraction, and Real-Time PCR

Enrichment of 226 samples, DNA extraction and PCR were performed as previously described (Le Maréchal et al., 2019).

### Multilocus Variable-Number of Tandem-Repeat Analysis (MLVA)

Thirteen DNA extracts positive for type D/C (with a Ct below 35) were selected for MLVA analysis using nine conventional PCRs (Supplementary Table 1), one for each variable number tandem repeat (VNTR) locus as described previously in Auricchio et al. (2019) and Scalfaro et al. (in preparation)^1^. The MLVA PCR mixture contained 10 μL of HotStarTaq Master mix (Qiagen, Courtaboeuf, France), 1 μL of forward and reverse primers (10 μM for a final concentration of 500 nM), 7 μL of water and 1 μL of template DNA. Amplification consisted of the following cycle program: 95°C for 15 min, 35 cycles of 30 s at 94°C, of 1 min at 55°C, of 1 min at 72°C, and one final cycle at 72°C for 5 min.

Multilocus variable-number of tandem-repeat analysis typing was performed on a T100 ThermalCycler (Bio-Rad, Marnes-la-Coquette, France). PCR products were analyzed by capillary electrophoresis using a 2100 bioanalyzer system (Agilent, Les Ulis, France) to determine the number of repeats for each VNTR locus, deduced from each PCR product size. Fragment length and the corresponding number of repeat units was also checked by sequencing on a 3500 Series Genetic Analyzer (Applied Biosystems, Thermo Fischer Scientific, Illkirch-Graffenstaden, France).

The number of repeats obtained from each VNTR locus was imported into Bio-Numerics version 7.5 (Applied Math, Sint-Martens-Latem, Belgium) as character values. A dendrogram was calculated using the categorical coefficient and the UPGMA clustering algorithm to compare DNA extracts from samples tested in our study and available profiles from a previous study (Auricchio et al., 2019) or from other animal botulism outbreaks (Supplementary Table 2).

## Results

### Chronology of the Events in the Mixed Farm

The chronology of the events is given in Table 1. Broiler Flock No. 1 was slaughtered on January 22, one week before the onset of the first clinical signs in cows. During their rearing period, an unusual event took place at 15 days of age: one side of the house collapsed due to a storm and caused the death of 120 broilers. The total mortality of this flock was 923 out of 20 000 broilers (4.6%). Otherwise, nothing unusual was reported regarding this flock nor for previous ones.

On January 26, poultry litter was removed from the house using a tractor bucket and manure was then stored in a field away from the farm (5 km from the farm). The same bucket was used later on the same day (after a quick rinse using a pressure cleaner with cold water) to prepare the cattle ration. Each ingredient of the cattle ration was taken one by one using the bucket and transferred to the mixing wagon to feed the cows later in the evening. Rape was taken first, then wrapped grass and meslin (all stored in Shed A as illustrated in Figure 1), then corn silage and finally brewery grains. Two days later, heifers were fed with the leftover dairy cattle ration. The day following the removal of poultry litter, Barn A was cleaned and disinfected as usual, with lime spread on the floor of the house and in the surroundings and a quaternary ammonium disinfectant sprayed (Spectragen^®^, Synthèse Élevage, France) in the house.

On January 29, three days after the distribution of the ration, paralysis and the first mortalities were noticed in the dairy cows in Barn B and the diagnosis of botulism type D/C was confirmed the next day. Two days later, on January 31, the same clinical signs occurred in heifers in Barn C.

The same farm personnel and equipment used for poultry and cattle were employed for both production units. Noteworthily, the poultry carcass bin was located next to the changing room of the poultry house and the rendering truck must drive by the cattle barns to reach the poultry house to collect poultry carcasses from the bin (Figure 1).

At the second cattle site 10 km away, no clinical signs were observed. This site was handled by the same farm personnel, using different equipment.

### Detection of *C. botulinum* Type D/C on the Farm

During the outbreak, 40 samples were collected at different times to investigate the source of contamination of cattle on the farm (Table 1). *C. botulinum* type D/C was detected in the five samples collected on February 5 from Flock No. 1 manure stored in a field since January 26 as well as three ingredients of the cattle ration (number of positive samples/number of samples tested, 3 + /17), i.e., meslin, wrapped grass, and rape. Wrapped grass was sampled at the same time as the samples for botulism diagnosis on January 30, whereas meslin and rape were sampled on February 5, several days after the initiation of botulism outbreak on the farm. Investigations conducted on February 23 revealed two positive samples in Barn B (liquid manure and swab of the floor), three positive samples in Shed A (swabs from the bucket, the mixing wagon and a rape tarpaulin) and three positive samples in the poultry house (two swabs from the ventilation system and one swab from the carcass bin). Given this detection of *C. botulinum* type D/C in the poultry house, extensive decontamination was implemented on March 19, with a strong cleaning agent (Decagen^®^ detergent, Synthèse Élevage, France), with disinfection (Virugen^®^, Synthèse Élevage, France) in the house and quicklime and caustic soda on the floor) and another disinfection using formaldehyde, with both procedures being carried out following the safety measures recommended when using such products.

After the outbreak, the 128 samples collected to monitor the contamination of broilers over time (Table 2) revealed the detection of *C. botulinum* type D/C in the ventilation system of the poultry house (8 + /14) (after the extensive disinfection of the house: 2 + /4; upon chick delivery of the following flocks, 4 + /8; and at the end of the rearing period, 2 + /2), contamination of some equipment coming from the hatchery (3 + /18) (2 swabs on chick box bottom and 1 swab of the egg transport trolleys and trolley wheels) and a continuous healthy carriage of broilers for several months detected in two consecutive flocks (4 + /20 and 17 + /20 cloacal swabs in two flocks and 1 swab of the floor litter). Moreover, the carcass bin was still detected positive after the extensive disinfection of the house.

### Detection of *C. botulinum* Type D/C in the Poultry Hatchery

Detection of *C. botulinum* at the hatchery is presented in Table 3. *C. botulinum* was detected in 6 out of the 58 samples: in the machine that cuts the article chick box, in the container of sludge washing water, on the loading platform, in the storage rooms of the uncleaned and cleaned egg trolleys, and on the wheels of the cleaned egg trolleys of hatching eggs. In addition, the epidemiological investigation revealed that the rendering truck entering the site to collect hatchery waste (silo of feathers and shells, breeder carcasses) passes hatchery vehicles or equipment (chick or egg delivery trucks, egg trolleys wheeled outside to be stored in annex rooms).

**TABLE 3:** Detection of *C. botulinum* type D/C at the hatchery delivering chicks on the farm (*n* = 58).

	Sampling location	Detection of *C. botulinum* type D/C
Inside the hatchery *n = 41	Egg receiving room Incubation room Transfer (to hatcher) room Hatcher room Changing room Chick sorting room Chick departure room	0 + /4 0 + /12 0 + /2 0 + /11 0 + /3 **1 + /5** Swab of the machine cutting the article box containing the chicks 0 + /4

vOutside the hatchery * *n* = 9	Trucks platforms Waste containers Central area Breeder house surroundings	**1 + /3**Swab of the loading platform **1 + /3**Swab of the container of sludge washing water 0 + /1 0 + /2

Annex room* *n* = 8	Wash room (uncleaned egg trolleys) Clean room (cleaned egg trolleys) Egg holding (cooler) room	**1 + /3** Swab of the room of the uncleaned egg trolleys (S9) **2 + /3** Swab of the room of the cleaned egg trolleys and swab of the trolley wheels (S10) 0 + /2

*Number of positive samples/number of samples collected. Samples tested by MLVA are underlined **and the sample code in**Figure 2 is indicated in **parentheses**. Samples detected positive are in bold.

### MLVA Analysis

Thirteen samples positive for *C. botulinum* type D/C were selected among the 291 samples analyzed in our study. Samples were selected so as to obtain an overview of the different investigated areas included in our study and to be as representative as possible of the encountered samples and situations, and finally to compare and evaluate the relatedness of strains detected in the different positive samples. Unfortunately, MLVA results obtained with samples initially detected positive for type D/C with a late Ct (above 35) using real-time PCR were not interpretable (such as for example the swab on the bucket).

Samples selected for MLVA analysis are indicated in Tables 1–3 and detailed number of detected repeats for each VNTR locus in Supplementary Table 3.

A dendrogram was generated based on the VNTR repeat unit profiles (Figure 2). Two different profiles were detected among the selected samples. Three cloacal swabs collected in Flock No. 4 presented a profile different from the other samples (76.8% of similarities between the two groups). These results show that at least two different strains were detected during these investigations with the detection in Flock No. 4 broilers with a profile different from the one detected during the cattle outbreak. However, no differences could be detected between the other investigated samples (Flock No. 1 manure, ruminal contents from a dead cow, cloacal swabs collected in Flock No. 3, samples from the hatchery), demonstrating the presence of the same strain in all these samples.

## Discussion

**FIGURE 3:**
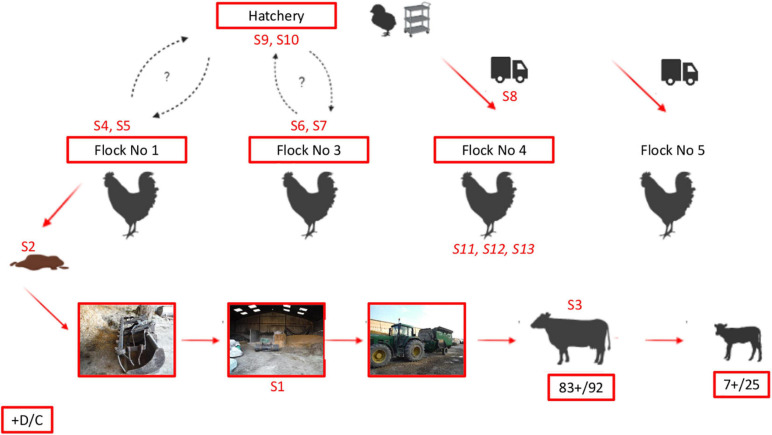
Diagram summarizing the likely relationships between the broilers, cattle, and hatchery based on investigations and results obtained in this study. Samples collected during the study and positive for *C. botulinum* type D/C are shown in red. References of samples tested using MLVA (Figure 2) are mentioned in the diagram. Note that samples S11–S13 are shown in italics in contrast to the other samples (S1–S10), because they showed a different MLVA profile (Figure 2). “+ D/C”: sample positive for *bont* D/C using PCR are surrounded in Red. Detailed results of samples collected and analyzed in our study are given in Table 1 (initial investigations during cattle botulism outbreak), Table 2 (monitoring of successive broiler flocks), and Table 3 (investigations in the hatchery that delivers chicks to the farm).

An overview of the detection of *C. botulinum* type D/C on the farm (poultry and cattle barns) and in the hatchery as well as the reference of samples that were compared using MLVA is provided in Figure 3. This figure also presents the most likely scenario of the contamination pathways suggested by our investigations.

### Identification of the Source of Cattle Contamination Through Epidemiological Investigations

Poultry manure has been reported to be a major source for cattle botulism outbreaks (Payne et al., 2011; Ramírez-Romero et al., 2014; Relun et al., 2017; Souillard et al., 2017). Poultry manure is also incriminated in this study, based on the chronology of the outbreak events, and our results showing positive samples (particularly the poultry manure samples) as well as MLVA profiles similar between the manure and the cows that suffered from botulism (Group 1, Figure 2). The positive manure samples from poultry Flock No. 1 (5 + /5) as well as the detection of *C. botulinum* in three samples collected within the poultry house (2 swabs of the ventilation system and 1 swab of the carcass bin) strongly suggest that *C. botulinum* type D/C was present in the poultry house when Flock No. 1 was present.

The tractor bucket used to remove broiler litter from the poultry house and then shortly after used to prepare the cattle ration was also positive for *C. botulinum* type D/C and may have transferred the contamination from poultry manure to the ration ingredients (3 + /17) and to the mixing wagon, which was positive as well. The three ingredients of the ration first taken with the bucket were all detected positive (rape, wrapped grass, and meslin). The MLVA results of the meslin moreover showed the same profile as Flock No. 1 manure and ruminal contents from a dead cow. The succession of these events seems therefore to have resulted in the contamination of dairy cattle fed with this ration, thereby initiating the outbreak. Ration leftovers were then distributed to heifers resulting in the onset of the disease in these animals as well. It is noteworthy that these samples were collected after the outbreak, except for the wrapped grass, therefore cross-contamination secondary to the outbreak cannot be completely ruled out. However, regarding the timing of the events and the analysis results, this seems unlikely. MLVA results also strongly support this scenario, the same profile being detected in Flock No. 1 manure, ruminal contents from a cow, ration ingredients as well as in samples collected in the poultry house after the Flock No. 1 rearing period.

An unusual event that occurred during the Flock No. 1 growth period may explain the manure contamination. The sudden death of 120 broilers due to the collapse of one side of the house during the rearing period may have increased the risk of carcasses being left in the litter despite daily carcass removal by the farmer. Carcasses as a decaying organic matter harboring high amounts of protein substrates and anaerobic conditions is known to support the growth of *C. botulinum* and BoNT production (Anniballi et al., 2013). Any stress factor in broilers can disturb the balance of the intestinal ecosystem (Tsiouris, 2016); therefore this event may have induced stress in broilers thereby providing favorable conditions for *C. botulinum* growth by modifying intestinal balance. The speed of the onset of clinical signs, 2 days after the distribution of contaminated ration, may be compatible with intoxication of the cattle by ingestion of preformed BoNTs. This hypothesis was supported by the detection of vegetative cells only, and not spores in the ruminal contents of two cows sampled at the beginning of the outbreak. Ruminal contents were only positive when analyzed using TPGY and not F-CMM, which includes a heat treatment.

In addition to this scenario, several biosecurity failures were also highlighted during the investigation. First, the storage conditions of the cattle ration ingredients were not appropriate, because they were stored uncovered, exposing them to potential contamination by wild birds or rodents. Providing safe, high-quality, and properly stored feed to animals is one of the key measures to minimize the risk of botulism (Anniballi et al., 2013). Rodents may be a reservoir of a variety of pathogens, in particular *C. botulinum* (Popoff, 1995; Popp et al., 2012; Skarin et al., 2013; Souillard et al., 2014). Moreover, the location of the rendering container (carcass bin), testing positive on two occasions, next to the poultry house can be a source of poultry contamination. Its location also obliges the rendering truck to drive alongside the cattle barns. Given the risk generated by carcasses in regard to botulism and the ability of spores to persist in the environment (Popoff, 1995), the management of the carcasses on a farm is a major critical point for the prevention of the disease. Third, equipment is also shared between the dairy and poultry production units, which can result in cross-contamination between the units. Finally, close proximity of poultry and dairy cows (less than 10 m between both barns with transversal ventilation in the poultry house) may also be a source of *C. botulinum* dissemination via dust and wind. Dust inside the ventilation system in the poultry house is frequently contaminated after a botulism outbreak (Souillard et al., 2014). Windborne transmission of spore- or BoNT- contaminated material has also been suggested (Hogg et al., 2008).

Since this outbreak, specific measures have been implemented to prevent recurrence of the disease: animals are vaccinated yearly, feed storage area has been reorganized, each component being now separated and protected; the bucket used to load feed in the mixing-wagon is now dedicated only to this activity, the tractor used for poultry manure is no longer used for cows feed; boots and clothes used for poultry and bovines by the farmer are now separated and dedicated to one activity. No new case has been reported since then.

### Monitoring of Broilers Contamination Over Time

Few data are available regarding healthy carriage of *C. botulinum* in poultry and it remains a current issue to better understand the onset of animal botulism (Rasetti-Escargueil et al., 2019). Our monitoring of broilers on this farm using cloacal swabs revealed continuous healthy carriage of *C. botulinum* in successive broiler flocks over several months. After the first detection of *C. botulinum* in the poultry house in January, the following broiler flocks remained healthy carriers of *C. botulinum* at least until August as demonstrated by positive cloacal swabs detected at the end of the rearing period for two flocks.

At least two scenarios can be considered to explain this persistent contamination of broilers over time on the farm. First, our monitoring showed persistence of *C. botulinum* type D/C in the house, particularly in the ventilation system (8 + /14), despite the disinfection operations conducted between each flock. This detection is likely due to the high resistance of spores in the environment that can survive for several years (Notermans et al., 1981; Wobeser et al., 1987), particularly in the critical areas of the poultry house that are difficult to disinfect, such as the ventilation systems, as reported previously (Souillard et al., 2014, 2016). Consequently, this environmental persistence of *C. botulinum* in the poultry house is a source of recontamination and may have resulted in healthy carriage in successive broiler flocks. A second scenario that may explain *C. botulinum* carriage in broilers for several months is the re-introduction of *C. botulinum* via chick delivery, suggested by the detection of *C. botulinum* on equipment from the hatchery. Two swabs collected on chick box bottoms for Flock No. 4 and one swab collected on trolleys and their wheels for Flock No. 5 were already positive for *C. botulinum* type D/C upon their arrival on the farm, before any contact with the farm. The same MLVA profile was detected for sample S8 (see Figure 2) as previous samples collected in the farm (Table 2 and Figure 3). Considering these findings, the question of a potential contamination of the hatchery thus arose and investigations were implemented in the hatchery delivering chicks to the farm to better explore this hypothesis.

### Evaluation of Hatchery Contamination

*Clostridium botulinum* type D/C was detected in 6 of the 58 samples collected in the hatchery: on materials (machine cutting the article box containing the chicks and wheels of trolleys transporting the hatching eggs), in the annex room (room where trolleys of hatching eggs are stored) and on outside surroundings (in the container of sludge washing water and on the platform where hatching eggs are unloaded). Contaminated areas were either located outside or in areas that are recognized as difficult to clean and disinfect. One sample positive for *C. botulinum* type D/C tested using MLVA showed the same profile (Group 1) as samples collected during the outbreak, demonstrating that the same strain was detected in both places.

During the hatchery visit and based on detection of *C. botulinum* type D/C in some samples, several risk factors were identified. First, similar vehicle routes were highlighted, in which rendering trucks and hatchery vehicles or equipment, for example, pass each other. Failures in cleaning and disinfection operations were also pointed out, as illustrated by the detection of *C. botulinum* type D/C on the system used to cut article boxes during chick delivery or in the room used to store cleaned trolleys or on wheels of cleaned trolleys.

In poultry breeding, the hatchery occupies a central position by being in daily contact with breeder farms to collect eggs and with broiler farms to deliver chicks. Hatcheries can serve as a reservoir and source of pathogenic microorganisms and via the movement of vehicles (delivery trucks), people or equipment (trays, trolleys, and chick boxes, etc.) can facilitate the dissemination of microorganisms (McMullin, 2009; Osman et al., 2018). Consequently, the contamination detected in our study in the hatchery can be also explained by this permanent, exchange of potentially healthy carriers of *C. botulinum* between the hatchery and broiler farms. It was not possible to identify the initial source of contamination, i.e., hatchery or poultry farms, here, because the hatchery was only taken into account after the botulism case investigation had begun.

Despite the hygiene and biosecurity procedures in the hatcheries, infectious agents can contaminate hatcheries by being transported on or within eggs, on hatchery personnel, on trolleys and trays, or as airborne contaminants (Wales and Davies, 2020). A wide range of microorganisms, such as *Salmonella, E. coli, Pseudomonas, Staphylococci, Mycoplasma*, or *Aspergillus*, can be detected in hatcheries and disseminated to chicks and subsequently to farms (Qureshi, 2002; McMullin, 2009). To the best of our knowledge, there is currently no data available regarding the contamination of hatcheries by *C. botulinum*. The risks of hatchery contamination arise from the hygiene of hatching eggs, the multiple exchanges between farms, and also the management of the hatchery, involving vehicles, people and equipment all along the process from the arrival of the hatching eggs to the delivery of chicks to customer farms (Qureshi, 2002; McMullin, 2009). In our study, *C. botulinum* type D/C was not detected in any of the samples collected in the incubation (*n* = 12) and hatching rooms (*n* = 11). Moreover, no asymptomatic carriage was detected in chicks delivered on the farm using cloacal swabs on Flock No. 5. The hatching process is considered as the most risky step for microbial dissemination (McMullin, 2009; Kim and Kim, 2010). This does not seem to be the case here based on detection results. No vertical transmission of *C. botulinum* from breeders to chicks was demonstrated here or in previous studies. The exact role of hatcheries in the epidemiology of animal botulism requires further investigation.

### MLVA as a Useful Tool for Epidemiological Investigations

Genotyping methods such as pulsed-field gel electrophoresis (PFGE) and amplified fragment length polymorphism (AFLP) have been successfully used in the context of animal botulism outbreaks for tracking strains (Myllykoski et al., 2009; Skarin et al., 2015). However such approaches require isolation of pure strains, thus limiting the number of samples that can be analyzed. Despite improvements regarding *C. botulinum* group III isolation (Le Gratiet et al., 2020), it is still time-consuming and phages encoding BoNT are easily lost. MLVA has been available for *C. botulinum* group I and II for many years (Fillo et al., 2011; Umeda et al., 2013; Anniballi et al., 2016). It has been recently developed for *C. botulinum* group III subtyping (Auricchio et al., 2019). MLVA presents a major advantage because it does not require the isolation of the strain and can be directly used on DNA extracted from the initial samples (Kahlisch et al., 2010; Chen et al., 2011; Pereyre et al., 2012; Pailhoriès et al., 2015). Our study demonstrates the usefulness of the MLVA approach as a subtyping tool intended for tracing and tracking *C. botulinum* group III, in particular for investigations during animal botulism outbreaks.

In our study, profiles of samples encoded S1 to S10 were shown to be identical (Group 1). Three cloacal swabs collected on broilers from Flock No. 4 presented a different profile from the other samples collected during the study (76.8% similarity between Groups 1 and 2). The origin of this second profile was not explained here. Unfortunately, MLVA results from samples collected at the beginning of the Flock No. 4 rearing period were not interpretable (DNA amount was too low in these samples as shown by a late Ct obtained for these samples). Therefore, it was not possible to determine if this contamination could be linked to the hatchery or not. Samples from the hatchery (samples S8, S9, S10) were indeed all part of Group 1.

## Conclusion

This study demonstrates that broilers can be healthy carriers of *C. botulinum* type D/C that can lead to cattle contamination and in the initiation of a botulism outbreak. This also shows that this contamination can last several months, spanning successive flocks. This study also shows that the environment of hatcheries can be contaminated by *C. botulinum* and may become a source of introduction of *C. botulinum* in poultry farms via chick delivery. As illustrated by a massive cattle botulism outbreak due to cross-contamination between poultry and cows, this study highlights the major importance of the implementation of appropriate biosecurity measures in mixed farms to avoid cross-contamination between the production units involving equipment (specific material or disinfection), vehicles (separate travel routes) and personnel (shoes and clothes in shower room). Further investigations are now required to evaluate *C. botulinum* contamination occurrence and level in hatcheries so as to better understand their potential role in the epidemiology of botulism.

## Data Availability Statement

The original contributions presented in the study are included in the article/Supplementary Material, further inquiries can be directed to the corresponding author.

## Ethics Statement

Samples were collected by veterinarians for diagnostic purposes on dead animals (death due to the botulism outbreak). Cloacal swabs were collected on broilers in the farm by veterinarians for official analyses. This article presents non-experimental clinical veterinary studies. Animals on farms were treated in accordance with the European and French regulation on farmed animal protection. Samples were collected in accordance with Regulation EC/1099/2009 and Directive 2010/63/EU. Written informed consent was obtained from the owners for the participation of their animals in this study.

## Author Contributions

CL, RS, SL, DG, and MC designed the study. CL, TP, SR, SM, and LM analyzed the samples for detection of *C. botulinum*. RS and CL analyzed the data and drafted the manuscript. DG, RS, and LB collected the epidemiological data and samples on field. TL and FA were in charge of the MLVA analysis. SL, FA, and MC provided their expertise feedback. All authors reviewed the draft and contributed significantly to the final manuscript.

## Conflict of Interest

The authors declare that the research was conducted in the absence of any commercial or financial relationships that could be construed as a potential conflict of interest.

## Footnotes

1 Scalfaro, C., Vicenza, T., Le Maréchal, C., Bano, L., Bilei, S., Chemaly, M., et al. (in preparation). Multilocus variable-number of tandem-repeat analysis as a tool for *Clostridium botulinum* group III sub-typing. *Microorganisms*
