# Supplementary material for: Asymptomatic Carriage of C. botulinum Type D/C in Broiler Flocks as the Source of Contamination of a Massive Botulism Outbreak on a Dairy Cattle Farm
Source: Front Microbiol. 2021 Jun 29;12:679377. doi: 10.3389/fmicb.2021.679377 (PMC8279769; doi:10.3389/fmicb.2021.679377)
Supplement: Supplementary file 1 [file Table_1.DOCX]

Supplemental Table 1 : List of primers used for MLVA analysis and repeat length of each VNTR locus

| VNTR Name | Forward Primer Sequence | Reverse Primer Sequence | Repeat length (bp) |
| --- | --- | --- | --- |
| MLVA_ANB_14 | CATATTTCCGCAGCCGTT | TGTGTTGAAGGCATGAATCC | 21 |
| MLVA_ANB_15 | TGGATCATGTGAAAATGCAAG | CAGCTCCTGCCTTCGCTA | 11 |
| MLVA_ANB_16 | GAAGCTGTAGGGCGTAGACAA | TGTCCTGGCATCAATTCTGA | 20 |
| MLVA_ANB_17 | TTTCTTCGTGCAATCCCTCT | GAAAATGCAAGGATGCTAAGA | 12 |
| MLVA_ANB_18 | TGTGAACCTGCCAACCCT | GGTGCACATTGACAACAGC | 9 |
| MLVA_ANB_19 | TTATCCGGGGTTCCCATT | CAACTTGATACCGGCCCC | 9 |
| MLVA_ANB_20 | GGACGCAACCTCAGAGAATG | ACTTCTTCGTCCATAGCTCCA | 15 |
| MLVA_ANB_23 | GGTTTAGATGCTAGAGGGTTTGA | AACGTGCTTCAAAAGGCAGT | 15 |
| MLVA_ANB_24 | CTGTTTCTCCCCTTTGTGTCA | GGCGTTAAAGGAAGCGGTAG | 18 |
