# Supplementary material for: Asymptomatic Carriage of C. botulinum Type D/C in Broiler Flocks as the Source of Contamination of a Massive Botulism Outbreak on a Dairy Cattle Farm
Source: Front Microbiol. 2021 Jun 29;12:679377. doi: 10.3389/fmicb.2021.679377 (PMC8279769; doi:10.3389/fmicb.2021.679377)
Supplement: Supplementary file 2 [file Table_2.DOCX]

Supplemental Table 2: Metadata of strains used to build the dendrogram (Fig. 2) and isolated from other animal botulism outbreaks in France.

| **Strain name** | **Origin** | **Year** | ***bont* type** | **French Department No** |
| --- | --- | --- | --- | --- |
| 20LNRB92.2 | Cattle | 2020 | D/C | 29 |
| 20LNRB49.3 | Cattle | 2020 | D/C | 29 |
| 20LNRB5.1 | Cattle | 2020 | D/C | 7 |
| 20LNRB83.3 | Cattle | 2020 | D/C, D, C, C/D | 22 |
| B19LNRB24 | Turkey | 2019 | D/C | 41 |
| 19LNRB12.1 | Guinea fowl | 2019 | D/C | 85 |
| 17LNRB7.2 | Cattle | 2017 | D/C | 56 |
