# Supplementary material for: Asymptomatic Carriage of C. botulinum Type D/C in Broiler Flocks as the Source of Contamination of a Massive Botulism Outbreak on a Dairy Cattle Farm
Source: Front Microbiol. 2021 Jun 29;12:679377. doi: 10.3389/fmicb.2021.679377 (PMC8279769; doi:10.3389/fmicb.2021.679377)
Supplement: Supplementary file 3 [file Table_3.DOCX]

Supplemental Table 3: Number of detected repeats for each VNTR locus

| **Sample name** | **VNTR14** | **VNTR15** | **VNTR16** | **VNTR17** | **VNTR18** | **VNTR19** | **VNTR20** | **VNTR23** | **VNTR24** |
| --- | --- | --- | --- | --- | --- | --- | --- | --- | --- |
| S1 Meslin | **0** | **0** | **2** | **0** | **6** | **0** | **1** | **2** | **2** |
| S2 Flock No. 1 manure | **0** | **0** | **2** | **0** | **6** | **0** | **1** | **2** | **2** |
| S3 Ruminal contents | **0** | **0** | **2** | **0** | **6** | **0** | **1** | **2** | **2** |
| S4 Swab of the carcass bin | **0** | **0** | **2** | **0** | **6** | **0** | **1** | **2** | **2** |
| S5 Swab of the ventilation system | **0** | **0** | **2** | **0** | **6** | **0** | **1** | **2** | **2** |
| S6 Cloacal swab (Flock No. 3) | **0** | **0** | **2** | **0** | **6** | **0** | **1** | **2** | **2** |
| S7 Cloacal swab (Flock No. 3) | **0** | **0** | **2** | **0** | **6** | **0** | **1** | **2** | **2** |
| S8 Swab of trolley and its wheels upon arrival | **0** | **0** | **2** | **0** | **6** | **0** | **1** | **2** | **2** |
| S9 Swab of the room for uncleaned trolleys | **0** | **0** | **2** | **0** | **6** | **0** | **1** | **2** | **2** |
| S10 Swab of cleaned trolley wheels | **0** | **0** | **2** | **0** | **6** | **0** | **1** | **2** | **2** |
| S11 Cloacal swab (Flock No. 4) | **0** | **4** | **2** | **0** | **6** | **0** | **2** | **2** | **2** |
| S12 Cloacal swab (Flock No. 4) | **0** | **4** | **2** | **0** | **6** | **0** | **2** | **2** | **2** |
| S13 Cloacal swab (Flock No. 4) | **0** | **4** | **2** | **0** | **6** | **0** | **2** | **2** | **2** |
